# Gender representation in leadership & research: a 13-year review of the Annual Canadian Society of Otolaryngology Meetings

**DOI:** 10.1186/s40463-023-00635-8

**Published:** 2023-05-11

**Authors:** Grace Yi, Jennifer Payandeh, Dorsa Mavedatnia, Penelope Neocleous, Jacob Davidson, Jennifer Siu, Molly Zirkle, Julie E. Strychowsky, M. Elise Graham, Yvonne Chan

**Affiliations:** 1 Department of Otolaryngology-Head and Neck Surgery, Temerty Faculty of Medicine, University of Toronto, St. Michael’s Hospital, 30 Bond Street, Unit 8CC-121, Toronto, ON M5B 1W8 Canada; 2 Faculty of Medicine, Queen’s University, Kingston, ON Canada; 3 Faculty of Medicine, University of Ottawa, Ottawa, ON Canada; 4 Schulich School of Medicine and Dentistry, Western University, London, ON Canada; 5 Division of Pediatric Surgery, Department of Surgery, Schulich School of Medicine and Dentistry, Western University, London, ON Canada; 6 Department of Otolaryngology-Head and Neck Surgery, Schulich School of Medicine and Dentistry, Western University, London, ON Canada

**Keywords:** Gender diversity, Women in otolaryngology, Leadership, Diversity, Equity, Inclusion, Panels

## Abstract

**Background:**

The gender disparity in surgical disciplines, specifically in speakers across North American medical and surgical specialty conferences, has been highlighted in recent literature. Improving gender diversity at society meetings and panels may provide many benefits. Our aim was to determine the state of gender diversity amongst presenters and speakers at the annual Canadian Society of Otolaryngology-Head and Neck Surgery (CSO) meetings.

**Methods:**

Scientific programs for the CSO annual meetings from 2008 to 2020 were obtained from the national society website. Participant name, role, gender, location, and subspecialty topic were recorded for all roles other than poster presenter. Gender (male or female) was determined using an online search. The total number of opportunity spots and proportion of women was then calculated. Gender differences were analyzed using chi-square test and logistic regression with odds ratios.

Four categories were analyzed: Society *Leadership*, Invited Speaker Opportunities, *Workshop Composition* (male-only panels or “manels”, female-only panels, or with at least one female speaker), and *Oral Paper Presenters* (first authors).

**Results:**

There were 1874 leadership opportunity spots from 2008 to 2020, of which 18.6% were filled by women. Among elected leadership positions in the society, only 92 unique women filled 738 leadership opportunity spots. 13.2% of workshop chairs, 20.8% of panelists and 22.7% of paper session chairs were female. There was an overall increase in the proportion of leadership positions held by women, from 13.9% of leadership spots in 2008 to 30.1% in 2020. Of the 368 workshops, 61.1% were led by men only, 36.4% by at least 1 female surgeon, and 2.5% by women only. “Manels” have comprised at least 37.5% of workshops each year.

**Conclusions:**

The proportion of women in speaking roles at the annual CSO meetings has generally increased over time, particularly among panelists, leading to fewer male-only speaking panels. However, there has been a slower rate of growth in the proportion of unique women in speaker roles. There remains an opportunity to increase gender/sex diversity at the major Canadian otolaryngology meeting.

**Graphical Abstract:**

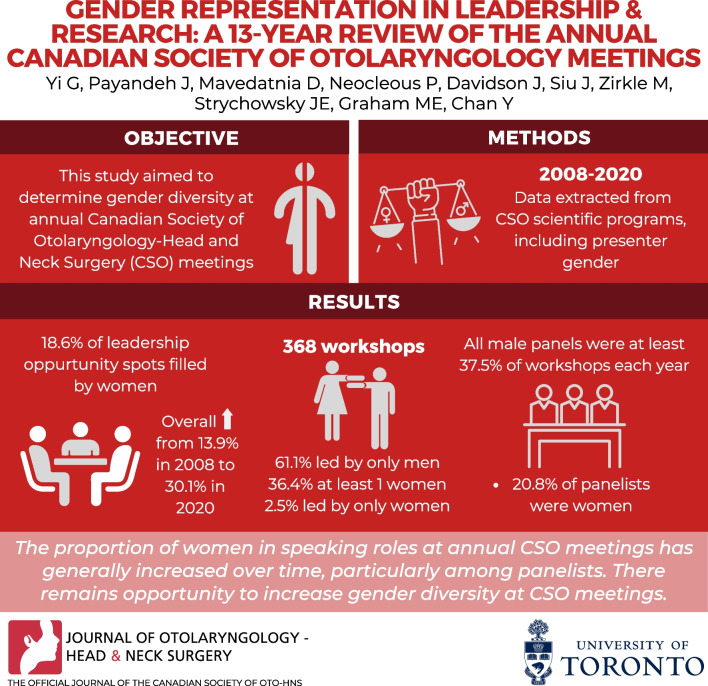

## Background

Gender disparity in surgical disciplines, including Otolaryngology-Head and Neck Surgery (OHNS), has been highlighted in recent literature. Over the past 20 years, the proportion of female staff otolaryngologists and trainees has increased by 14.2% and 13.3% respectively, where 24.2% of staff otolaryngologists were female, and 41.9% of residents were female as of 2019 [1, 2]. Despite these advances, women lack proportionate representation in leadership positions in OHNS academic departments and specialty societies, though this may be improving among junior academic positions [3–5].

Termed “manels”, male-only speaking panels at major scientific conferences have been a recent focus in the literature. Women speakers were underrepresented across multiple medical and surgical specialty conferences, including in cross-sectional analyses of various American and Canadian society meetings [6–10]. In 2019, Nature Conferences and Springer Nature released a new code of conduct to formalize efforts to increase gender diversity, including no male-only organizing committees, no male-only panels, annual monitoring of progress, and sanctions when the code is not followed [11]. Dr. Francis Collins, the National Institute of Health director, stated that women and other minorities were not equitably represented at major scientific conferences. He vowed to help “end the Manel tradition” by refusing to speak at a conference if attention to diversity was not given [12].

Diversity in society meetings and panel-type presentations has multiple benefits. It has the potential to expand perspectives and several studies have shown that varied opinions may lead to better ideas, innovation, and an overall stronger panel [13]. Women physicians have been shown to provide stellar patient care with excellent outcomes and have a place on these panels [14–17]. Increasing equitable representation of women and others helps perpetuate to attendees that individuals of all backgrounds are important members of the specialty society. Presentation at academic meetings and participation on scientific panels is also important for career advancement in academia. The presence of female representation helps decrease the “glass ceiling” effect noted for women in academia [13, 18]. Finally, this is an issue of justice and inclusivity [19].

While there have been studies on gender diversity amongst speakers at key surgical conferences in the United States and Europe, there has not been published literature assessing this in our specialty in Canada. The Canadian Society of Otolaryngology-Head and Neck Surgery (CSO) is the major Otolaryngology society in Canada and encompasses all Otolaryngology subspecialties. Our aim was to determine the state of gender diversity amongst presenters and speakers at the annual CSO meetings.

## Methods

Scientific programs for the CSO Annual Meetings were obtained from their website (www.entcanada.org) from 2008 to 2020 by two independent groups of researchers at two Canadian institutions. Extracted information for each position included: participant name, gender, role, and subspecialty topic (General OHNS, Education, Laryngology, Pediatric, Otology, Head and Neck Surgery, Facial Plastics and Reconstructive Surgery (FPRS), Endocrinology, and Rhinology). CSO annual newsletters were also accessed to extract the name and gender of CSO executive leadership. A binary definition of gender (male or female) was chosen as a surrogate of diversity in the study population, composed of specialists trained in Otolaryngology, Otolaryngology trainees, other medical specialists, allied health members, and medical students. Gender was determined using an online search of Google Scholar, departmental websites, and public descriptions. If gender could not be determined from online information, co-authors and fellow panelists were contacted to determine this information.

### Leadership

CSO Executive Membership was extracted from CSO annual newsletters and included members of the executive council, executive committee, regional representatives, and special interest group leaders. Each of these was defined as a leadership “opportunity spot” and the number of unique women occupying these roles was quantified. To quantify the degree of diversity, each position was counted as one “opportunity spot” as per Barinsky et al. [18] to capture those who participate in several different roles.

### Invited speaking opportunities

An invited speaking opportunity spot was defined as any named role in the CSO program other than paper session or poster presenter (i.e. session moderator). Of the opportunity spots occupied by a woman, the absolute number of women included was also assessed. The following roles were included: CSO president, scientific program chair, local arrangements chair (if provided), guest(s) of honour, guest speakers and special presenters, award winners, workshop presenters and panelists, and paper session chair.

### Composition of panels

The composition of panels was separately analyzed and divided into male-only panels, female-only panels, or those with at least one female participant. The CSO meetings labelled sessions led by one or a small group of experts as “mini-workshops”, “workshops”, “courses” or “panels”. Among workshops with multiple presenters, those with two or fewer presenters were named “workshop chairs”, and those with three or more presenters were called “panelists”. Those who were designated “workshop chairs” with a separate panel were named as “workshop chairs”. All named non-otolaryngologists (including other medical specialists, allied health specialists, researchers) and non-Canadian otolaryngologists were included in the count.

Descriptive statistical analysis was performed using SAS Software (Version 9.4, SAS Institute Inc., Cary, NC, USA), and consisted of counts and percentages. The two data sets produced by the two independent groups were merged into a single file to cross-check. A senior author (EG) reviewed the file and flagged any inconsistencies in the data, which were then investigated and corrected based on publicly available information. The senior author identified 48 errors (approximately 4%), which were corrected. Gender differences were analyzed using chi-square tests and logistic regression with odds ratios (OR) and 95% confidence intervals (95%CI). An alpha level of 0.05 was used to determine statistical significance. This project did not require ethics oversight as per article 2.2. of the Tri-Council Policy Statement (TCPS)-2 guidelines regarding the use of publicly available data for research purposes.

## Results

**Fig. 1:**
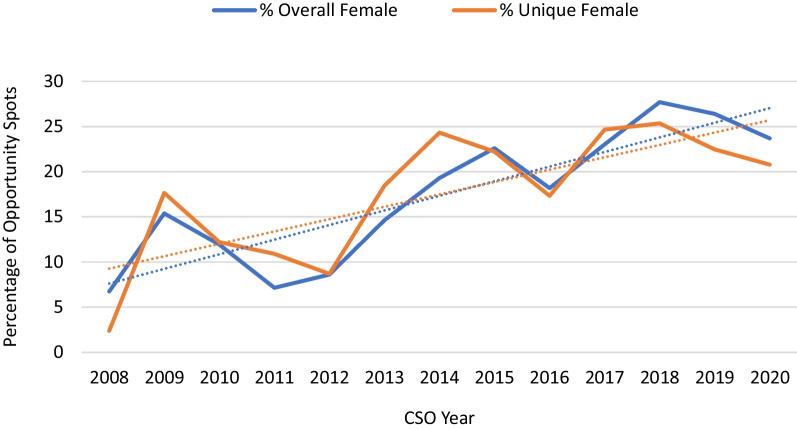
Proportion of leadership opportunity spots filled by women at Canadian Society of Otolaryngology meetings: 2008–2020. The proportion of women filling leadership opportunity spots and the proportion of unique women filling these spots are presented. The dotted lines represent an upward linear trend in the proportion of females in leadership opportunity spots over time. The rate of growth in the absolute number of women occupying leadership opportunity spots is similar to the proportion of women occupying these spots

A total of 1874 opportunity spots were available during the annual CSO meetings from 2008 to 2020, of which 348 (18.6%) were filled by women (Table 1). These were held by 92 unique women in total. There was an overall increase in the number and proportion of these positions held by women (Fig. 1), from six leadership spots in 2008 (6.7%) to a peak of 50 spots in 2020 (23.7%).

**Table 1:** Summary and Overview of Gender Representation at Canadian Society of Otolaryngology meetings: 2008–2020

Variables	Males	Females
	% (n)	
**CSOHN leadership roles**	**n = 601**	**n = 137**
Session moderator	77.3 (153)	22.7 (45)
Executive council	87.2 (82)	12.8 (12)
Special interest group	91.2 (145)	8.8 (14)
Executive committee	75.7 (140)	24.3 (45)
Regional representative	79.4 (81)	20.6 (21)
**Invited speaking opportunities**	**n = 925**	**n = 211**
Workshop	81.4 (79)	18.6 (18)
Panels	81.4 (846)	18.6 (193)

### Leadership

**Fig. 2:**
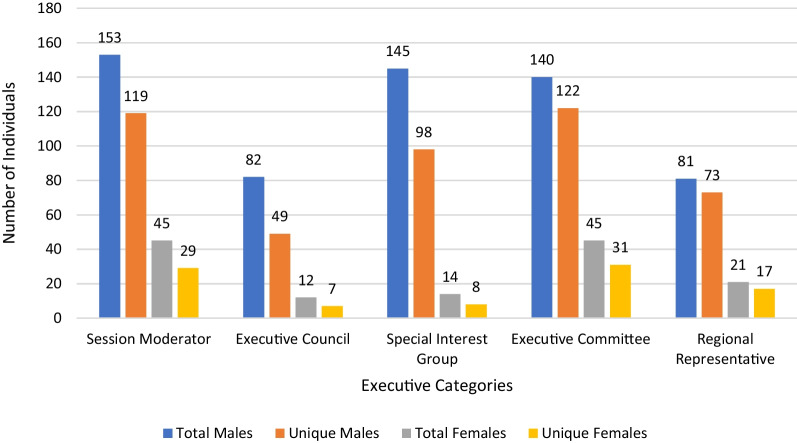
The gender distribution amongst Canadian Society of Otolaryngology executive leadership positions: 2008–2020. Males represented a greater number of total and unique opportunity spots on executive counsels compared to females. This remains consistent across each executive category

**Fig. 3:**
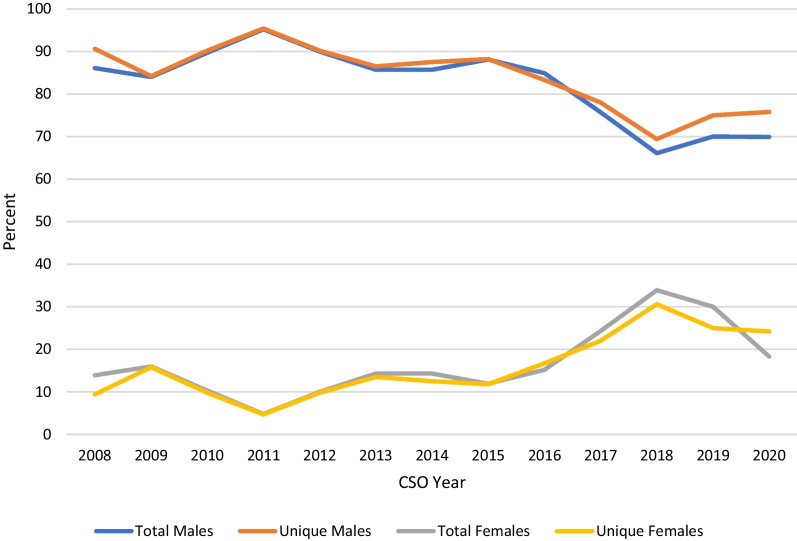
The trend in the number of men and women occupying Canadian Society of Otolaryngology executive positions: 2008–2020. A greater proportion of males occupied executive positions within the CSO between 2008 and 2020. The proportion of positions held by female members has shown a gradual increase over time

Among all CSO executive members, there were 448 men (83.0%) and 92 women (17.0%) over the studied period, encompassing 342 unique men and 63 unique females. The gender breakdown by position type, along with the number of unique individuals occupying these positions, is shown in Fig. 2. There was a significant difference between male and female representation in society executive members (p = 0.0009). Figure 3 shows the change in gender representation in executive positions over the period studied, with the trend in unique individuals occupying these positions. Notably, there has been one female CSO president, and no female scientific program chairs during the period studied. Among the Guests of Honour, of which there are usually one or two per meeting, there has been only one female otolaryngologist chosen across all meetings. The CSO Awards Committee Chair, who also serves as chair of the annual Poliquin competition for resident research, has been a male surgeon until 2019 and 2020, when a female surgeon was elected to this role. From 2011 and 2014 onwards, various awards were given for lifetime achievement, recognition by Canadian region, and fellowship awards. Of the thirty-one awards, seven (22.6%) were awarded to women across all years studied.

### Invited speaking opportunities

**Fig. 4:**
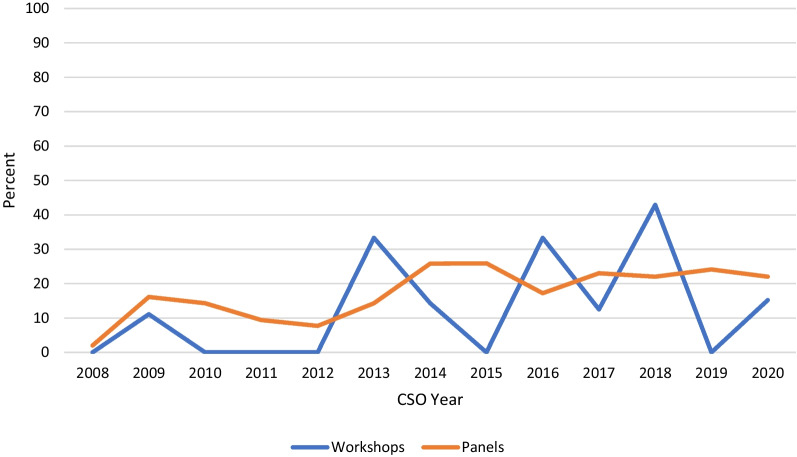
Proportion of female representation in workshops and panels at Canadian Society of Otolaryngology meetings: 2008–2020. The proportion of female representation in CSO panels has remained relatively constant since 2014. There are no discernable patterns in female representation in CSO workshops

Overall, there were 1,136 invited speaking opportunities at CSO meetings between 2008 and 2020. Of these, 97 were part of workshops and 1,039 from panels. Females only represented 18.6% (18) of invited speakers at workshops and 18.6% (193) at panels. Across each CSO year, female representation in panels steadily increased until 2015, and then has remained constant at around 20 to 25% (Fig. 4). There appears to be no discernible trend in female representation in workshops over the same period.

The Scientific Program Committee consists of the CSO president, Scientific Program Chair, and Continuing Professional Development (CPD) Committee Chair. In the period studied, this committee included one woman (of 3–4 members) in 2008, 2013, and from 2015–2020. The larger Scientific Program Reviewer Committee consisted of 20–25 members representing all OHNS subspecialties, who reviewed blinded abstracts for selection of workshop/panel presenters, oral session presenters, and poster presenters. Data was available for 2018–2020 only, and there were seven female members in 2018, seven in 2019, and five in 2020.

### Composition of panels

**Fig. 5:**
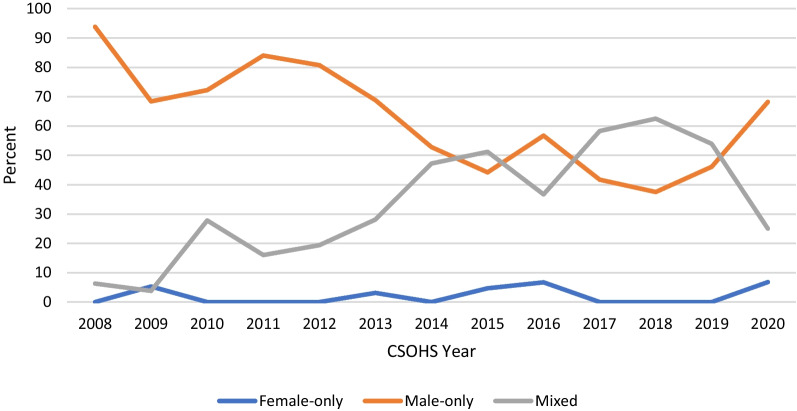
Proportion of sessions led by men only (“manels”), women only, and by one or more woman (“mixed”) at Canadian Society of Otolaryngology meetings: 2008–2020. The term “sessions” includes all groups of multiple speakers. There has been no considerable change in the proportion of female-only sessions between 2008 and 2020. However, it does appear that mixed sessions have overall increased, whereas male-only presentations have decreased over time

A total of 368 workshops (including workshops, mini-workshops, panels, courses, and CPD Corner sessions) were identified. There were 225 (61.1%) male-only panels (“manels”), while 9 (2.5%) were led by women only, and 134 (36.4%) workshops included at least one female surgeon (Fig. 5). Chi-square analysis showed a significant difference between the proportion of male-only panels and those including any women (p = 0.0001). The CSO meeting in 2015 was the first year that there was a greater proportion of panels including at least one woman than those with exclusively male panelists (55.8% mixed panels), and this trend has continued for four out of six subsequent years.

Female leadership was significantly underrepresented in many subspecialties (Table 2). Conversely, laryngology and general OHNS workshops consistently had more female representation, even from 2008. There were only five instances, between 2008 and 2020, where females made up the majority of representatives in their discipline’s sessions compared to their male counterparts.

**Table 2:** Proportion of sessions that included women by subspecialty at Canadian Society of Otolaryngology meetings: 2008–2020

CSO year	General	Rhinology	Head and neck surgery	Pediatrics	Otology	Laryngology	FPRS*	Endocrinology	Education
	% (n)								
2008	0.0 (0)	0.0 (0)	0.0 (0)	0.0 (0)	0.0 (0)	25.0 (1)	0.0 (0)	0.0 (0)	0.0 (0)
2009	22.2 (2)	0.0 (0)	50.0 (1)	0.0 (0)	9.1 (1)	40.0 (2)	0.0 (0)	0.0 (0)	0.0 (0)
2010	6.7 (1)	9.1 (1)	14.3 (1)	0.0 (0)	0.0 (0)	30.8 (4)	0.0 (0)	0.0 (0)	0.0 (0)
2011	0.0 (0)	20.0 (2)	0.0 (0)	0.0 (0)	5.0 (1)	60.0 (3)	0.0 (0)	0.0 (0)	0.0 (0)
2012	13.9 (5)	0.0 (0)	10.0 (1)	0.0 (0)	0.0 (0)	20.0 (1)	0.0 (0)	0.0 (0)	0.0 (0)
2013	18.6 (8)	25.0 (1)	33.3 (3)	0.0 (0)	6.3 (1)	0.0 (0)	0.0 (0)	0.0 (0)	0.0 (0)
2014	30.8 (12)	14.3 (2)	14.3 (2)	0.0 (0)	30.8 (4)	40.0 (4)	0.0 (0)	0.0 (0)	0.0 (0)
2015	20.8 (10)	13.3 (2)	16.7 (1)	33.3 (1)	37.5 (9)	50.0 (6)	19.1 (4)	33.3 (2)	0.0 (0)
2016	24.3 (9)	6.3 (1)	0.0 (0)	50.0 (6)	0.0 (0)	22.2 (2)	0.0 (0)	20.0 (1)	0.0 (0)
2017	31.0 (9)	8.3 (1)	0.0 (0)	60.0 (3)	50.0 (2)	50.0 (4)	0.0 (0)	33.3 (2)	0.0 (0)
2018	27.1 (16)	9.1 (1)	16.7 (1)	0.0 (0)	0.0 (0)	75.0 (3)	0.0 (0)	0.0 (0)	0.0 (0)
2019	37.5 (18)	0.0 (0)	27.8 (5)	0.0 (0)	14.3 (3)	0.0 (0)	0.0 (0)	0.0 (0)	0.0 (0)
2020	37.0 (10)	4.4 (1)	14.0 (8)	100.0 (1)	0.0 (0)	55.7 (5)	0.0 (0)	0.0 (0)	0.0 (0)

^*^Facial, Plastics, and Reconstructive Surgery

All workshops/panels that included at least one woman were included in the count. Percentages only show the proportion of women members of each workshops/panels by subspecialty for each CSO year

## Discussion

Our data demonstrated that female surgeons held nearly a quarter of the total speaking positions at the CSO meetings from 2008 to 2020. The most common roles held were paper session chairs and panelists (a workshop led by three or more specialists). The proportion of male-only panels and workshops (“manels”) did decrease over time, but constituted over half of all workshops in 2020. Our results align with the current literature highlighting the differential representation of women in academic conferences, particularly in medicine and in surgical subspecialties [6–10, 20, 21].

Barinsky et al. were the first and only group to publish on the gender disparity of OHNS conference speakers in the US. They showed an increase in opportunity spots occupied by women from 11.5% in 2003 to 29.5% in 2019, but that the number of unique women occupying these spots was only 24.4% of the total [18]. Women were more likely to be oral session moderators or panelists instead of speakers, executive board members, or honoured guests. This was mirrored in our results in the Canadian population. It is promising that we have seen a trend toward increasing female representation over the past 12 years, especially amongst workshop chairs and panelists. Part of this may be attributed to an increase in the number of female otolaryngologists in Canada, from only 10% in 2000 to 24% in 2019 [1], which does approximate the proportion of female speakers in those years. An increase in female representation in leadership positions was seen starting in 2014. We hypothesize that there may be several contributing factors—the opening of more opportunities to present workshops, a critical mass of female staff and trainees moving through the pipeline, and the development of a formal Women in Otolaryngology section of the CSO.

“Mini-workshops” and “How I Do It” workshops were first introduced at CSO meetings in 2014, though they were not always present in subsequent years. The increase in opportunities, particularly of smaller workshops, may be a way of increasing opportunities for participation from more junior staff, a pool of specialists more likely to include women [2]. Our results also showed increased female representation in broader subspecialties starting in 2014. The proportion of Canadian and American women pursuing academic fellowships in surgical specialties has increased over the past several decades [1, 22]. From 2011 to 2020, the number of Canadian female otolaryngologists who have completed subspecialty fellowships has increased. Still, the gender gap was largest in head and neck surgery, rhinology, and otology, where only 28%, 29%, and 22% were female, respectively. Pediatric OHNS and laryngology were the only two fellowships with a female predominance. However, the absolute number of female graduates of otology, rhinology, and facial plastic surgery ranged from 5–10 over 2011 to 2020, whereas the numbers of female graduates of head and neck surgery and pediatric OHNS were similar at 15–20, and more than 30 new general OHNS practitioners were female [1]. This correlates with our findings that there was less female representation in facial plastics and rhinology workshops.

Increasing mentorship opportunities and visibility of women and minorities can lead to increased participation in academic activities by junior staff and trainees [13, 23, 24]. The CSO Women in Otolaryngology (WIO) group was established in 2014, and coincides with the increased female presence at the annual meeting. The WIO hosts networking sessions with female staff and trainees from across the country and offers opportunities for society leadership, mentorship, and creates a sense of community. This may be critical for incoming and junior trainees navigating transitions and seeking career advancement opportunities. Deliberate initiatives such as this will continue to raise awareness of gender disparities in our specialty, and encourage females to pursue academic aspirations, an essential first step toward increasing representation.

In 2019, 41.9% of OHNS trainees were female, and 45.3% of OHNS CaRMS applicants were female, indicating that future generations may see greater gender parity. We expect to see a similar trend in our speakers and conference leadership as more women become involved in academic endeavours. Literature shows that despite increasing proportions of female trainees and surgeons, women are still underrepresented in OHNS leadership and senior academic roles (such as assistant, associate, and full professor) compared to men [5, 25–28], and when compared to all specialties in medicine [29, 30]. However, a lag effect may be contributing to this phenomenon, in that it will take several years for the newly admitted trainees to eventually progress through their careers to leadership positions. To close the gender disparity amongst conference speakers and presenters, there must be continued efforts to close the gender gap among trainees entering the specialty, increase support for women to pursue research and academia [31], and develop initiatives to recruit and retain female faculty [25].

Studies from Arora et al., Lu et al., Gerull et al., and Zaza et al. assessed the proportion of female speakers at an aggregate of over a hundred academic medical and surgical conferences across multiple specialties. They examined the correlation between the proportion of women on conference planning committees and female speakers [9, 10, 20, 21]. There was a statistically significant positive correlation between the proportion of women on planning committees and society leadership and the proportion of female speakers, based on univariable analysis and still significant after controlling for regional gender balance of the specialty. For our study, the scientific planning committee information was only fully available from 2018 onwards, and while it would have been interesting to support this literature with our study, this analysis was not possible in a meaningful way. Increasing the proportion of women on conference planning committees may be a simple yet effective way to reduce the gender disparity amongst speakers [9, 10, 13, 20, 21].

Our conference has a blinded selection process, with workshop chairs and presenters submitting blinded abstracts to be selected by the scientific planning committee. The gender disparity in workshops may not be related to gendered selection bias, but rather the number of women conducting research and their research productivity. While a 2013 study reported that women in their early career produce less research output, but at senior levels, they equal or exceed the research productivity of men [32], a more recent report from 2020 indicates that female otolaryngologists are maintaining research productivity in their early careers (less than 15 years into practice) to keep closer pace with men. However, women continued to lag behind men in research productivity in some subspecialties such as head and neck oncology, laryngology, and pediatrics [33]. There are likely numerous contributing factors affecting research productivity, but the evolution of societal gender roles with more equal sharing of domestic duties and child care, greater financial and administrative support for research, and increasing mentorship opportunities will have a positive impact [19, 23, 31, 33].

This study is only one component in achieving greater equity and diversity: raising awareness of disparities. Moving forward, we must consider systems-level change to improve gender parity 11, 31]. It is critical to further assess the factors impacting speaker invitations for conferences, and women’s submissions for these opportunities. These may include personal and professional barriers, the proportion of women in the specialty, research productivity, visibility as a leader in the field, gender bias, and gender composition of the conference planning committee 9, 10, 13, 20, 21]. Regular reassessment of female representation at these conferences is a crucial checkpoint 18]. Ongoing analysis of equity at national society and departmental levels may be facilitated by designated diversity and inclusion leads or committees, and including these stakeholders in conference and departmental planning [25]. With the higher proportion of women amongst younger otolaryngologists and trainees, continuing to improve the gender gap will result in a larger pool from which to select our conference leadership and presenters.

### Study limitations

The results of the study must be interpreted within the confines of the research methodology. This study is limited in that it is a retrospective review of various publicly available databases, and thus the authors were unable to confirm the accuracy or validity of this data. Data around the proportion of abstracts submitted by female presenters versus the proportion accepted for presentation was not available. We also used a binary definition of biological sex as a surrogate for gender identity, which exists on a spectrum, and the biological sex of presenters was recorded based on public information and/or confirmation by colleagues. Lastly, the present study did not capture the many other diversity factors in the workforce.

## Conclusion

The proportion of women in speaking roles at the annual Canadian Society of Otolaryngology-Head and Neck Surgery meetings has generally increased with time, particularly among panelists. This has led to a decrease in male-only speaking panels and workshops. However, there has been a slower growth rate of unique women in leadership speaker roles. There is still room for increasing gender diversity at the major Canadian OHNS meeting. Academic mentorship, equitable allocation of opportunities and resources, and equal encouragement of research endeavours for both men and women may help contribute to this.

## Data Availability

The datasets generated and/or analyzed during the current study are available on the Canadian Society of Otolaryngology-Head and Neck Society website. It is also available from the primary/corresponding author on reasonable request.

## Abbreviations

OHNS: Otolaryngology Head and Neck Surgery
CSO: Canadian Society of Otolaryngology- Head and Neck Surgery
FPRS: Facial Plastics and Reconstructive Surgery
WIO Group: Women in Otolaryngology Group
TCPS-2: Tri-Council Policy Statement-2
CPD Committee Chair: Continuing Professional Development Committee Chair
